# Design and testing of fabric-based portable soft exoskeleton glove for hand grasping assistance in daily activity

**DOI:** 10.1016/j.ohx.2024.e00537

**Published:** 2024-05-07

**Authors:** Rifky Ismail, Mochammad Ariyanto, Joga D. Setiawan, Taufik Hidayat, Paryanto, Limbang K. Nuswantara

**Affiliations:** a Depertment of Mechanical Engineering, Faculty of Engineering, Diponegoro University, Semarang, Indonesia; b Center for Biomechanics Biomaterials Biomechatronics and Biosignal Processing (CBIOM3S) Diponegoro University, Semarang, Indonesia; c Professional Education of Engineers, Faculty of Engineering, Diponegoro University, Semarang, Indonesia; d Graduate School of Engineering, Mechanical Engineering Department, Osaka University, Suita, Japan

**Keywords:** Soft glove, Fabric-based, Hand grasping, Motor-tendon, Assistive device

## Abstract

Hand exoskeleton robots have been developed as rehabilitation robots and assistive devices. Based on the material used, they can be soft or hard exoskeletons. Soft materials such as fabric can be used as a component of the wearable robot to increase comfortability. In this paper, we proposed an affordable soft hand exoskeleton based on fabric and motor-tendon actuation for hand flexion/extension motion assistance in daily activities. On-off control and PI compensator were implemented to regulate finger flexion and extension of the soft exoskeleton. The controllers were embedded into a microcontroller using Simulink software. The input signal command comes from the potentiometer and electromyography (EMG) sensor to drive the flexion/extension movement. Based on the experiments, the proposed controller successfully controlled the exoskeleton hand to facilitate a user in grasping various objects. The proposed soft hand exoskeleton is lightweight, comfortable, portable, and affordable, making it easily manufactured using available hardware and open-source code. The developed soft exoskeleton is a potential assistive device for a person who lost the ability to grasp objects.

Specifications table

**Table:** 

Hardware name	Fabric-Based Portable Soft Exoskeleton Hand
Subject area	• Engineering • Medical (rehabilitation robotics) • Educational tools and open-source alternatives to existing infrastructure
Hardware type	• Mechanical engineering • Robotics
Closest commercial analog	*Active Hand Exoskeletons*
Open source license	*Creative Commons Attribution-ShareAlike 4.0 International License (CC BY-4.0)*
Cost of hardware	*307.35 $*
Source file repository	*https://doi.org/10.5281/zenodo.7542701*

## 1 Hardware in context

The development of robotic hand exoskeletons in the medical field is progressing rapidly. The primary function of the wearable hand exoskeleton robot in the medical world is as a rehabilitation tool and assistive device for patients with hand paralysis such as stroke, spinal cord injury (SPI), or brachial plexus injury (BPI). [1], [2], [3]. Researchers have developed hand exoskeletons using hard/rigid material rigid or flexible linkage for rehabilitation [4], [5], [6], [7] and assistive devices [8], [9], [10], [11]. Researchers developed 3D printed exoskeleton for lightweight, easy customization, and affordable manufacturing cost [12], [13], [14], [15].

Researchers developed a soft hand exoskeleton that is more comfortable to wear and easier for users to use. Due to its flexible nature, the soft hand exoskeleton can prevent or minimize the potential for injury when used and operated by the user. Based on the actuators used, most soft hand exoskeletons implement pneumatic networks, tendons based using electric motor, and smart memory alloy (SMA). Researchers created an exoskeleton to help stroke survivors use soft materials to increase strength without the downside of using stiff materials by restricting a person's natural movements. The soft exoskeleton gloves were developed using a pneumatic network [16], [17], [18], [19], [20] and hydraulic actuators [21], [22]. Fiber-reinforced components generate bending motion to assist the finger with flexion and extension. Pneumatic and hydraulic are utilized in soft exoskeleton actuators due to high efficiency and power transmission, but they need maintenance (fluid leakage) and generate noise [23]. As another alternative, researchers employ a tendon system (electric motor and SMA) as the actuator because it is cheaper and needs less maintenance. This system drawback is low power transmission efficiency from the actuator to the soft glove. Fabric and silicone are the commonly used materials for pneumatic and hydraulic hand exoskeletons. Fabric-based exoskeleton generates force capability higher than silicone-based ones [24].

The actuator system for motor-tendon and SMA can be more compact than pneumatic and hydraulic networks. Silicone rubber and polymer were used as the base material for exoskeleton gloves with motor-tendon actuators [25], [26], [27], [28]. SMA-based soft actuators have been used to drive the soft robotic glove [29], [30], [31], [32]. The soft glove system using motor-tendon and SMA actuators can be designed in compact form. SMA provides a higher force/weight ratio compared to pneumatic networks and electric motors, but it is more challenging to control because of its complex thermal phase transformation [33].

This paper proposes an affordable fabric-based soft hand exoskeleton glove driven by motor-tendon actuation. Using this system, a comfortable, portable, lightweight, and easy-to-manufacture soft glove can provide mechanical assistance for people who lost the ability to grasp objects. A linear actuator with position feedback was chosen as the main actuator to drive the soft glove. SR10 fabric was selected as the main material of the soft glove. Proportional-Integral (PI) control was embedded in the soft glove microcontroller for regulating the motion of finger flexion and extension using Simulink Support Package for Arduino Hardware. A study participant wore and utilized the developed soft glove for grasping assistance in daily activity for various object grasping. A user could provide a signal command to the soft glove using a wireless remote control or electromyography (EMG) sensor.

## 2 Hardware description

**Fig. 1:**
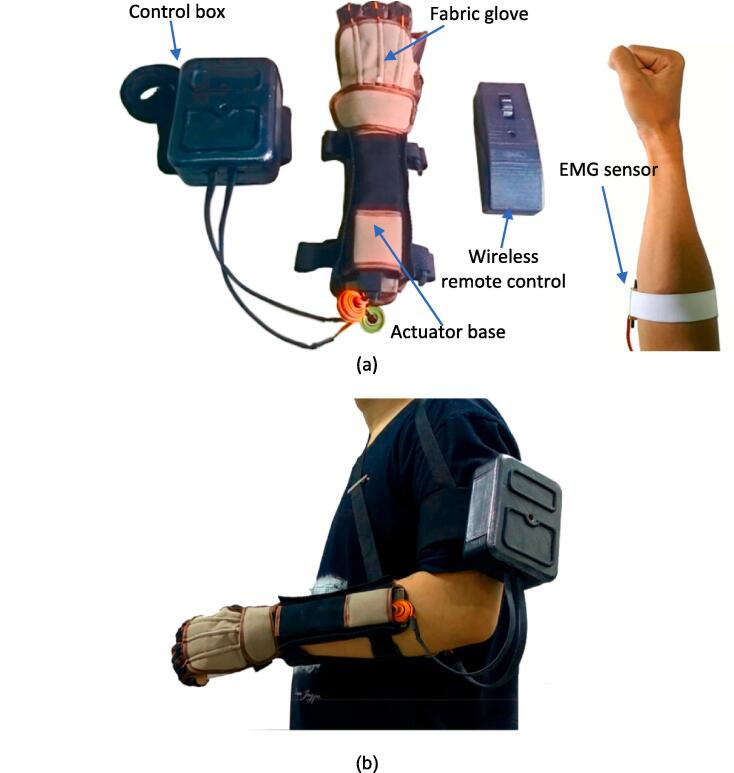
Fabric-based soft exoskeleton glove (a) Soft glove system (b) Portable soft glove when worn by a user.

The soft glove consists of four main components: the glove, actuator base, controller box, and sensor (remote control/EMG sensor). The fabric-based soft robotic glove parts are shown in Fig. 1(a). The glove and actuator base are attached to the hand of the person with paralysis, the controller box is attached to the user's upper arm/shoulder. While the remote control can be held by the user's normal and healthy hand. Soft gloves must have a high level of comfort when used because these tools will be used in daily activities with a long duration of use. This study showed that this soft glove was quite comfortable to use for a long time. This soft glove does not burden the user's body too much because the total weight of this tool is only 600 g. The semi-open shape of the glove does not make the wearer's hands sweat as much as when wearing fully closed gloves. Then the operation of the tool is quite easy to do, namely, to move this soft robotic glove, the user simply rotates the potentiometer/button on the wireless remote control. The actuator in this soft glove functions as a tendon puller to perform flexion and extension movements of the fingers. The Soft glove provides mechanical assistance for the index, middle, and ring. Meanwhile, the user's thumb is fixed at a fixed angle and does not move. By implementing this, soft glove can provide grasping assistance for a user to do daily activities.

The soft glove is driven by a linear actuator where to move the actuator, a microcontroller, and other electrical components are needed which regulate and give orders to the actuator to make a move. So we need a container to put these electrical components in one place which is then named as a control box. In this study, input commands from the potentiometer are used to give motion commands to the actuator, which are located separately from the controller box that regulates the actuator motion. Remote control consists of a microcontroller and a potentiometer as command input. Therefore, it is necessary to design a controller box and remote control. Factors to be considered in making an electrical case, namely in terms of size and shape. Control box mounted on the shoulder. The design of the controller box is compact and lightweight so that it does not burden the hand and interferes when moving, likewise, with the remote control design used. The remote control design is compact and easy to operate. In this study, the electrical casing and remote control were designed with a simple external form and tended to be easy to manufacture but still paying attention to the aspects mentioned above.

Motor-tendon type actuator was selected and implemented in a soft glove. The motor-tendon actuator system will reduce the dimensions of the glove so that a compact soft exoskeleton glove can be made. In the motor-type actuator tendon actuator, a rope is needed to function as a pulling tool that works like a muscle tendon in the organs of the human body. The rope/tendon on the motor-actuator tendon is used to pull the fingers so that the knuckles can bend and elongate. For this reason, several things need to be considered in choosing the tendon material to be used, namely ease of installation, lifetime, strength, and size. Nylon was chosen as the link between the actuator and the soft glove because of its ability to withstand large pulls, is strong, is quite durable, small in size, and is easy to install. This nylon tendon is used as a link between the hooks on the knuckles and the linear actuators that pull and stretch the movement of the fingers.

Soft gloves have a high level of comfort when used so that users can use them for daily activities with a long duration of use. The glove does not weigh too much on the wearer's body because the weight of the control box and the soft glove is only about 600 g, excluding the remote and EMG sensor. The semi-open shape of the glove does not make the wearer's hands sweat as much as when wearing fully closed gloves. The operation of the soft glove system is quite easy. To operate this soft glove system, the user only needs to turn the potentiometer on the wireless remote control or attach EMG to the muscles of the hand, which are still functioning normally. The components of the soft glove system can be obtained easily on e-commerce. This robotic glove system can be manufactured easily, quickly, and programmed without high-level knowledge of programming languages like C++. The specifications of the proposed soft glove can be summarized in Table 1.

**Table 1:** Specifications of the portable fabric-based soft glove.

Index	Specification
Glove material	SR10 fabric
Wight of soft glove	360 g
Weight of controller box	240 g
Weight of remote control	129 g
Supported fingers	Index, middle, ring
Actuators	Two linear actuators with position feedback
Communication range	<100 m (open space and lower baud rate)
Microcontroller unit	Arduino Nano (ATmega328)
Supported force (max.)	6 N
Finger speed from extension to full flexion	1.9 s
Flexion/extension control	Proportional-Integral (PI)
Operating voltage	12 V

## 3 Design files summary

The firmware for the wireless remote and control box was not embedded on the Arduino Nano microcontrollers using Arduino IDE. Instead, Simulink Support Package for Arduino Hardware was chosen for the embedded system. The toolbox can be freely downloaded and installed under MATLAB/Simulink software. It is a graphical programming language for the embedded control system. Therefore, it is easier for someone with insufficient low-level programming skills, such as C++. The embedded firmware in the SLX format for the control box (PID_remote_wireless.slx, and PID_EMG.slx) and wireless remote (Remote_wireless.slx) is provided In Table 2. The embedded firmware for the control box and wireless remote is presented in Section 5.3.

**Table 2:** Design File name and summary.

Design file name	File type	Open source license	Location of the file
BOM_exoskeleton_glove.xlsx	Excel	CC BY-4.0	https://doi.org/10.5281/zenodo.7542701
Fabric-based glove sketch	JPEG	CC BY-4.0	https://doi.org/10.5281/zenodo.7542701
Control box (lid, top, base box)	CAD files	CC BY-4.0	(Folder: CAD controller box) https://doi.org/10.5281/zenodo.7542701
Wireless remote (lid, top, wheel, remote base)	CAD files	CC BY-4.0	(Folder: CAD remote) https://doi.org/10.5281/zenodo.7542701
PID_remote_wireless.slx(firmware on the control box)	Simulink program	CC BY-4.0	(Folder: Simulink control box) https://doi.org/10.5281/zenodo.7542701
PID_EMG.slx(firmware on the control box)	Simulink program	CC BY-4.0	(Folder: Simulink control box) https://doi.org/10.5281/zenodo.7542701
Remote_wireless.slx(firmware on the remote wireless)	Simulink program	CC BY-4.0	(Folder: Simulink remote) https://doi.org/10.5281/zenodo.7542701
Movie Soft Exoskeleton Glove.mp4	Video (mp4)	CC BY-4.0	https://doi.org/10.5281/zenodo.7542701

## 4 Bill of materials summary

Bill of material summary is provided in Table 3 which contains the detail of the components, quantity, cost of the components and the its source.

**Table 3:** Bill of materials.

Designator	Component	Number	Cost per unit −currency	Total cost − currency	Source of materials	Material type
Battery	3.7 V 18650, 3000 mAh	4	5.38	21.52	AliExpresshttps://www.aliexpress.com/	Electronics
Tendon	Monofilament Nylon Fishing Line 100 *m*	1	0.97	0.97	AliExpresshttps://www.aliexpress.com/	Material
Glove material	SR 10 water-resistant fabric	3	3.29	9.87	Shopeehttps://shopee.com/	Material
3D printer filament	PLA Filament 1 kg	1	8	8	AliExpresshttps://www.aliexpress.com/	Material
Microcontroller unit	Arduino Nano 16 MHz, ATmega328	2	2.23	4.46	AliExpress https://www.aliexpress.com/	Electronics
Actuator	Linear actuator with potentiometer feedback L16-P 100 mm	2	80	160	Actuonixhttps://www.actuonix.com/l16	Electronics
Switch	Push Button Switch 10x15mm SPST 2Pin	2	0.73	1.46	AliExpress https://www.aliexpress.com/	Electronics
Potentiometer	Linear Potentiometer 15 mm Shaft With Nuts and Washers 3pin	1	0.29	0.29	AliExpress https://www.aliexpress.com/	Electronics
Wireless module	Nrf24l01 + Wireless Data Transmission Module 2.4 g	2	0.63	1.26	AliExpress https://www.aliexpress.com/	Electronics
Actuator driver	L293D DIP-16 Chip Driver Dual Motor	1	0.71	0.71	AliExpress https://www.aliexpress.com/	Electronics
Force sensor	Force Sensitive Resistor Thin Film Pressure Sensor 20 g-1.5 kg	3	1.49	4.47	AliExpress https://www.aliexpress.com/	Electronics
DC-DC switching buck converter	DC-DC 5–32 V Adjustable Step Down 5A Buck Power Supply Module	1	1.35	1.35	AliExpress https://www.aliexpress.com/	Electronics
Voltage regulator	LD1117V3 (two) and LM7812 (one) Voltage Regulator	3	0.83	2.49	AliExpress https://www.aliexpress.com/	Electronics
Tendon hose	Silicone Rubber Hose (inner diameter 1.5 mm and outer diameter 1.9 mm)	1	1.5	1.5	Alibabahttps://www.alibaba.com/	Material
Sewing thread	Polyester Durable sewing Knitting Thread	1	1.58	1.58	AliExpress https://www.aliexpress.com/	Material
Electromyography sensor	Myoelectric electromyography Sensor Muscle Electrical Signal Sensor	1	87.42	87.42	AliExpress https://www.aliexpress.com/	Electronics

## 5 Build instructions

### 5.1 Fabric-based soft exoskeleton glove instruction

**Fig. 2:**
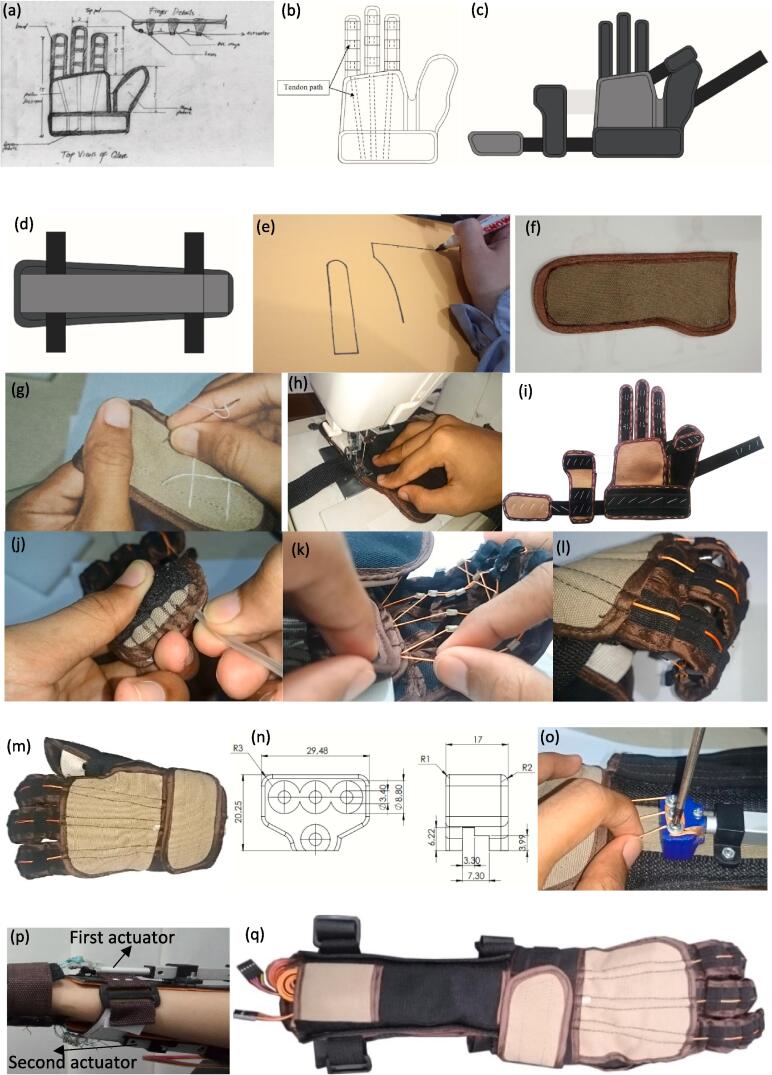
Soft glove and actuator base manufacturing (a) Soft glove sketch (b) Tendon path (c) Soft glove base design (d) Actuator base design (e) Drawing pattern on SR10 fabric (f) Hand sewing (g) Glove part (h) Machine sewing (i) Fabric glove (j) Silicone rubber attachment (k) Tendon installation on the finger (l) Tendon route on the finger (m) Soft glove with tendon (n) Padding tune design (o) Actuator and tendon connector using padding tune (p) Attaching two linear actuators (q) Soft glove with tendon and actuator.

The application of a soft robotic exoskeleton is relatively new for wearable robots. In this study, the main material for making gloves is fabric. Therefore, it is necessary to design the basic shape of the glove (base glove). When viewed from a functional point of view, the base glove will become a blanket for the knuckles and palms, wherein there is a tube for the tendon pathway. The base glove's shape will affect the soft glove's performance and aesthetics. The final design sketch is shown in Fig. 2(a). It was chosen because the thumb and glove cross sections have been combined in this design but do not cover the entire hand like gloves in general. This design is intended to make the glove easier to attach and detach. This makes air circulation smoother so that users do not sweat quickly when using gloves for a long time, but the stiffness of the gloves is still good. In addition, the wrist straps are wider to hold the glove position when used, so it does not shift easily. In the process of making a soft glove, several things need to be considered, namely the fingers and the place where the tendons are placed on the glove. The tendon path in the soft glove is made by inserting a tube in the glove. The tendon path connecting the actuator and the finger glove is shown in Fig. 2(b).

The primary material of the soft robotic glove is fabric. It was chosen because it is soft when in contact with human skin, absorbs sweat, is easy to clean, and comfortable to wear like a piece of clothing we often wear. In order for the soft glove to work properly and comfortably when it is used, it is necessary to select a fabric material that meets certain criteria, namely stiffness, thickness, comfort, and price. The SR 10 type fabric material was chosen as the soft glove material. The properties of this material are water-repellent, heat-resistant, stain-resistant, and strong (tear-resistant). This type of cloth is very suitable for outdoor purposes. In addition, the SR 10 fabric has a texture that is quite soft on the hands and is a thick material when compared to cotton and jeans. When viewed from the comfort of the material when used, cotton is the most comfortable because this material is soft, cool, and absorbs sweat easily compared to SR 10-type fabrics and jeans. However, cotton has the disadvantage of being easily torn because of its thin material. Jeans-type fabrics are stiffer and thicker than cotton or SR 10 fabrics. However, because the texture of the cloth is quite rough when used as gloves, where the cloth will often rub against the skin, it will cause blisters on the skin and can cause irritation. As for the SR 10 type fabric, this fabric has a fairly dense fabric texture. Therefore, it makes this cloth not easily torn and has a soft texture, so it is best used as a glove material in this study. The design of the fabric-based soft glove is presented in Fig. 2(c).

The actuator base, which contains the actuator can be attached to the user's hand and does not cause fatigue when operated. Therefore, it is necessary to design an actuator base where to place the linear actuator as the driving motor so that it does not easily shift when the actuator pulls the tendons to move the actuator base's hand on the soft robotic glove. The actuator base is designed close to where the glove is located to facilitate the tendon pulling mechanism as shown in Fig. 2(d). In this study, the actuator base was placed on the arm below the elbow to the wrist to align the linear actuator with the glove. The actuator base is made of cloth which is then inserted inside with a Polyvinyl chloride (PVC) plate to make it stiff.

In the soft glove manufacturing process, the way to make a glove is by sewing it. The basic steps that must be taken at this stage are to design a stitch pattern, make a pattern on paper, make a pattern on the fabric, and then cut the fabric according to the pattern that has been made. The parts that have been made need to be joined by sewing manually and finally by combining all the parts of the fabric pattern into one whole so that the desired glove is formed using a sewing machine so that it is strong. Fig. 2(e) shows the process of making a stitch pattern on paper.

Making a pattern on a soft glove is quite an essential stage because, at this stage, we determine the basic shape of the glove to be made, including the fingers, palm, shell, and actuator base. Making a pattern on paper will make it easier to modify the size or make gloves of the same size next time. Then the next step is to draw a pattern on the cloth used and then cut it according to the shape of the pattern made. The results of cutting the fabric that has followed the pattern are shown in Fig. 2(f).

After finishing cutting all the fabrics according to the pattern, the next process is to unite the fabric parts by sewing manually. This process uses a sewing needle and thread to create rough stitches. This stage aims to place the glove parts properly and prevent the fabric from shifting easily when sewn using a sewing machine. The manual sewing process is shown in Fig. 2(g). The thing that must be considered when sewing manually is that the needle size selection must be adjusted to the thickness of the fabric. For thick fabrics, use a large needle as well. In addition, in order to facilitate the work process, we should choose a thread color that is different from the color of the fabric to be used so that you can avoid mistakes when sewing with a sewing machine and it is easy to remove the rough stitches.

The final shape of the gloves is visible in the manual sewing process. However, the rough seam from the workmanship is not very strong if the soft glove is used in the long term. Therefore it is necessary to strengthen the stitches on the gloves with a sewing machine. With a sewing machine, the stitches will be neat and strong. Before using the sewing machine, we must determine the parts that require tight stitches and loose stitches. If all the seams used are tight, it will make the gloves wrinkle, making them uncomfortable when used. On the other hand, if all the seams use loose stitches, there will be parts that come loose and come off easily. The sewing process using a sewing machine is shown in Fig. 2(h), and the final sewn soft glove is shown in Fig. 2(i).

After all the parts on the gloves are sewn correctly, the next step that must be done is the stage of attaching the hose to the gloves. This study used a hose with an inner diameter of 1.5 mm and an outer diameter of 1.9 mm. The hose material is made of silicone which is quite flexible but retains its shape well so it doesn't make the glove stiff after inserting a hose inside. The main purpose of inserting the hose in the glove is as a pathway for the tendon so that the tendon does not unravel, which can cause abrasions if it rubs against the skin and so that the tendon does not get stuck due to contact with the glove fabric. In addition, this hose makes the shape of the gloves sturdier and does not bend easily. The process of attaching the hose to the glove is shown in Fig. 2(j).

The parts inserted into the soft glove hose include the knuckles, palm, and part of the palm. The hose is inserted according to the previously sewn space. To simplify the process of installing the hose, a tool in the form of a small wire rod is used. Then the size of the hose should not come out too much from the glove because it will make the hose easily slide out. The next step is to attach the tendon to the glove where the tendon path covers the knuckles, palm, and ends at the actuator base. The process of attaching the tendons is shown in Fig. 2(k).

The installation of tendons is grouped into two parts, namely, the upper tendon and the lower tendon. The lower tendon will later be connected to the actuator, which is located at the bottom of the hand as well as the upper tendon. The results of the glove manufacturing process are shown in Fig. 2(l). After the entire sewing process is complete, the glove is checked again whether there are parts that have not been sewn properly or if there are any loose stitches. If there is residual suture thread sticking out, it is necessary to cut it and to prevent the stitches from falling back due to the thread earlier, and the thread is burned using a gas lighter carefully so as not to burn other parts. The glove sewn and attached to the tendons is shown in Fig. 2(m).

Tendons made of nylon were chosen because they are strong, and flexible but a bit slippery when the tendons are attached to something. Obstacles when attaching the tendon to the actuator occur due to the slippery nylon when attached to the actuator. The nylon thread ties easily shift or even come off the actuator when moving the glove. To overcome this problem, additional components are made in the form of tuning pegs on a guitar which function is almost the same, namely to adjust the length of the nylon thread tied to the actuator while simultaneously tightening the bond between the nylon thread and the actuator. The material used to make these tuning pegs is PLA, with the dimensions shown in Fig. 2(n). These tuning pegs have three pegs made of 3 mm diameter bolts locked using nuts. The number of pegs is adjusted to the number of fingers to be moved, namely the index finger, middle finger, and ring finger so that we can adjust the length of the nylon thread on each finger.

To adjust the length of the tendons on the soft robotic glove prototype, loosen the pegs, adjust the length as needed, and then tighten the pegs again. The process of tightening the pins on the tuning pegs is shown in Fig. 2(o). The final step is to connect the actuator base with the sewn glove. The first linear actuator is attached to the lower arm next to the palmar side of the hand, while the second actuator is placed to the lower arm next to the dorsal side of the hand, as depicted in Fig. 2(p). Both actuators are covered with sewn SR10 fabric. The results of a soft glove integrated with the actuator base are shown in Fig. 2(q).

### 5.2 Control box and wireless remote build instruction

**Fig. 3:**
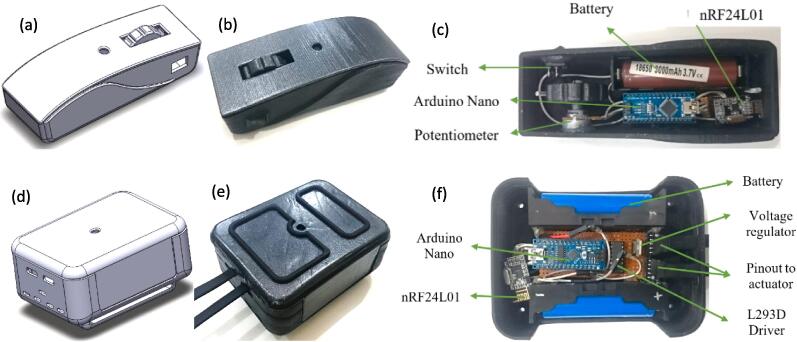
Wireless remote and control box (a) Wireless remote CAD design (b) Wireless remote (c) Wireless remote with electronic components (d) Control box CAD design (e) Control box (f) Control box with electronic components.

The soft glove is driven by a linear actuator where to move the actuator, a microcontroller, and other electrical components are needed which regulate and give orders to the actuator to move. A container is needed to put these electrical components in one place, called the control box. To give motion commands to the actuator, input commands are used from the potentiometer/EMG sensor which is located separately from the controller box, which regulates the actuator motion. We decided to make a wireless remote control that consists of a microcontroller and a potentiometer as command input. Therefore, it is necessary to design a controller box and remote control. Factors to be considered in making an electrical case, namely in terms of size and shape. Control box mounted on the shoulder. The controller box's design is compact and lightweight, so it does not burden the hand and interferes when moving. Likewise, with the remote control design used. The remote control design is compact and easy to operate. We make electrical and remote control casings with simple external shapes and tend to be easy to manufacture but still pay attention to the aspects mentioned above. The first step is to design the casing by hand sketching. To tidy up the design and for manufacturing purposes, the results of the hand sketches must be translated into CAD drawings. SolidWorks is chosen to create CAD designs for electrical casings and remote controls. All aspects of the design, including the dimensions of the casing and the positioning of the electrical hardware, are directly outlined in this CAD drawing. The CAD controller box and remote control can be seen in Fig. 3(a) and Fig. 3(d).

After the CAD controller box and remote control designs were finished, the manufacturing process for both devices was carried out. We made control box and remote control forms in this study using a 3D printer. The material used is PLA (Polylactic Acid). PLA is one of the materials commonly used to make electronic device casings with a melting point of 210 °C, and it is lightweight as the base material. After the controller box and remote control have been printed, the finishing process is then carried out by smoothing the rough parts on the surface and the parts where the electrical components are placed. In addition, a painting was carried out to beautify the appearance of the remote control and controller box. The final results of the controller box and remote control can be seen in Fig. 3(b) and Fig. 3(e).

After all the electrical components that will be used have been obtained, the next step is to create an electrical circuit schematic on the project board. The Fritzing software is selected to make an overview of the project board. In this study, there were two electrical circuits: the transmitter electrical circuit (wireless remote) and the receiver electrical circuit (soft glove). One 18,650 Lithium Ion (Li-ion) battery with a capacity of 3000 mAh is applied to power the wireless remote. This transmitter circuit functions to read the potentiometer and send it wirelessly to the soft glove. In this transmitter box, a potentiometer provides an input signal that regulates the linear actuator movement. Meanwhile, a wireless module is used to transmit data that utilizes the 2.4 GHz radio wave band, namely NRF24L01.

**Fig. 4:**
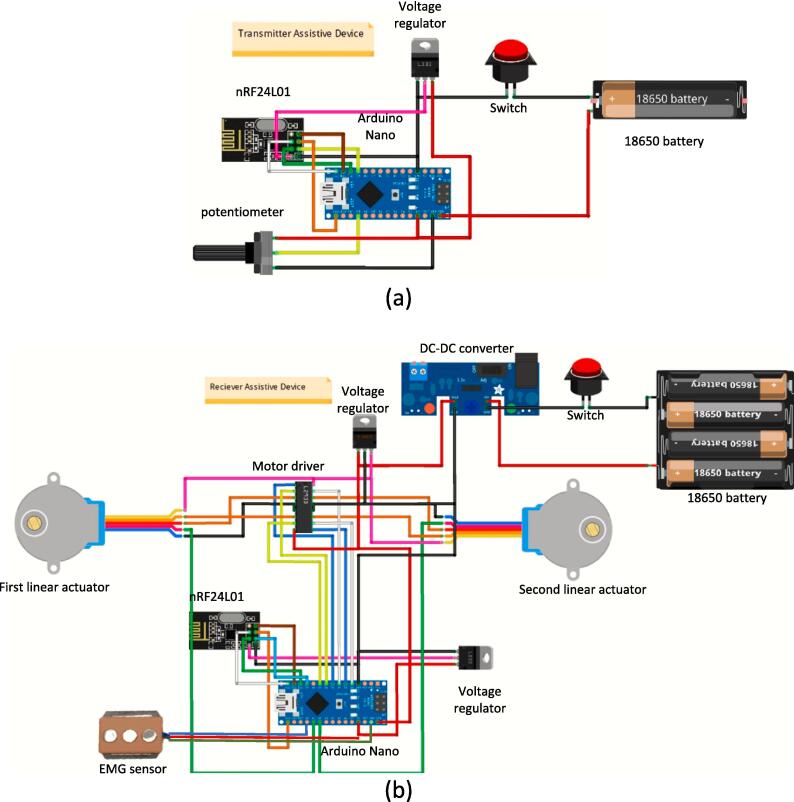
Fritzing schematic diagram of (a) Wireless remote (b) soft exoskeleton glove.

Two low-drop linear voltage regulators from STMicroelectronics (LD1117V3, TO-220 package) were selected to step down the 3.7 V from a Li-ion battery to output a constant voltage of 3.3 V. This voltage output was utilized to provide the voltage input to the wireless NRF24L01 module in the wireless remote and soft exoskeleton glove as shown in Fig. 4. An adjustable DC-DC switching buck converter was applied to reduce the voltage from four 3.7 V 18,560Li-ion batteries connected in series. The output from the DC-DC converter was regulated at 12 V. A step-down linear voltage regulator (LM7812, TO-220 package) was applied to provide a fixed regulated output voltage of 12 V DC from a DC-DC converter. This input voltage was implemented for both linear actuators in the soft exoskeleton glove. This 12 V DC can provide an optimal response for both linear actuators in the closed-loop system. For details on connecting the wireless remote circuit and the soft glove receiver, see Fig. 4(a) and Fig. 4(b).

### 5.3 Embedded control

**Fig. 5:**
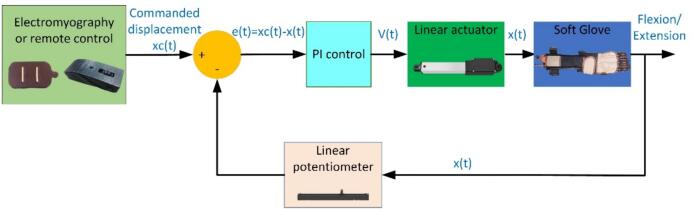
Embedded proportional-integral (PI) control on soft exoskeleton glove.

The last step of building a soft robotic glove system is implementing a control system and embedding it into the microcontroller. Arduino Nano was chosen as the central controller for the soft glove because of its small size and can be programmed easily using MATLAB/Simulink software. Free toolbox from MATLAB, Simulink Support Package for Arduino Hardware is selected as the main embedded software due to its easy embedded system development and graphical programming language for the embedded control system. Proportional-integral (PI) control is implemented to control the stroke length of the actuator x(t) and provide mechanical support for finger flexion/extension. PI control used in this soft glove aims to get good control of the stroke position of the actuator as desired by a user. The selected Kp and Ki are 8.5 and 0.005 respectively. The control input for the linear actuator can be calculated using Equation (1), and the magnitude of the voltage is determined using Equation (2). The overall block diagram for PI control on the soft glove is presented in Fig. 5. The control signal u(t) or the provided voltage V(t) is determined by the proportional gain (Kp) multiplied by the error e(t), with the addition of the integral gain (Ki) multiplied by the integral of the error. The error e(t) is determined by calculating the difference between the commanded displacement, xc(t), and the measured displacement of the linear actuator, x(t).

(1) u(t)=V(t)=Kp×e(t)+Ki×∫e(t)dt

(2) $$V\left(t\right)=\left\{\begin{array}{c} V,e(t)\geq 0\\ -V,e(t)<0 \end{array}\right)$$

**Fig. 6:**
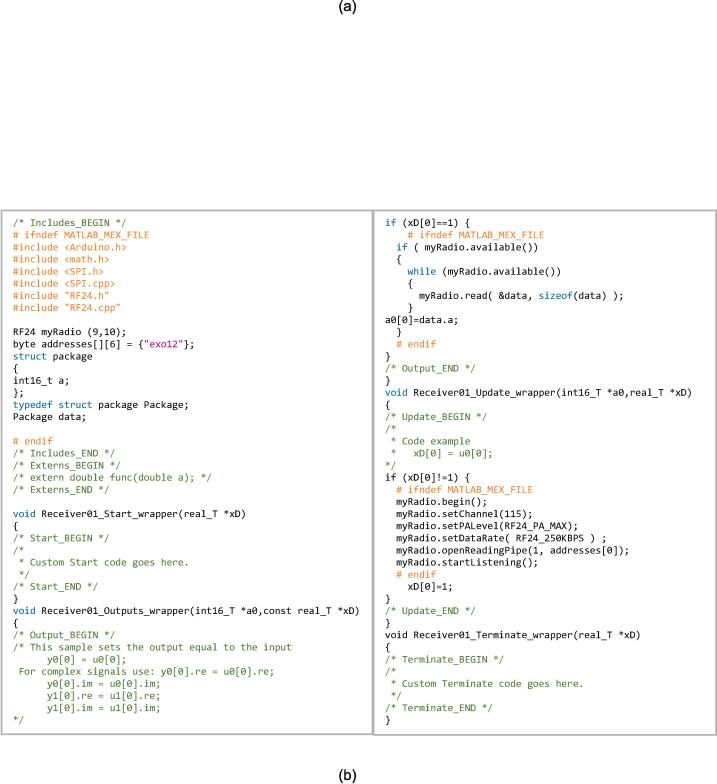

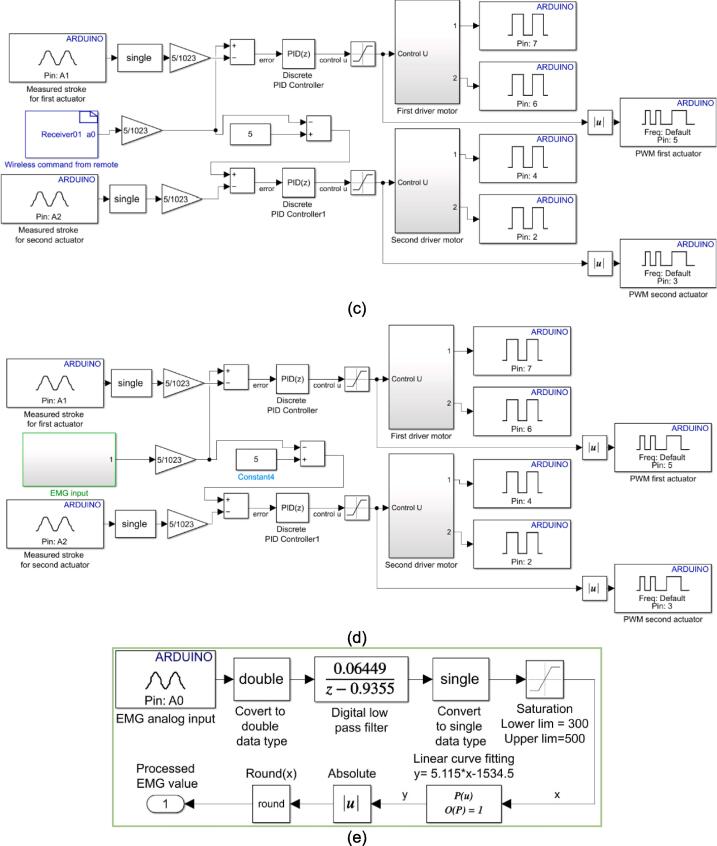
Proposed embedded system for soft exoskeleton glove (a) Wireless remote block diagram (b) S-Function block program for wireless transmitter (c) PI control block for exoskeleton with wireless remote (d) PI control block for exoskeleton with EMG sensor (e). EMG input block for processing the output signal.

A block diagram of the wireless remote “Remote_wireless.slx”, an analog input block is used to read the potentiometer sensor, as shown in Fig. 6(a). A custom block diagram to send the potentiometer value is done by writing C++ code in the S-Function block. The code for transmitting the potentiometer value is presented in Fig. 6(b). Two sensors are used in the embedded control soft glove: the potentiometer and EMG. The block diagram of the wireless remote with a potentiometer “PID_remote_wireless.slx” is shown in Fig. 6(c). The linear potentiometer contained in the linear actuator is used to read the stroke length.

Two analog input blocks read analog values from two linear actuators. The PID control block is used to implement the PI control written in Equation (1). Meanwhile, the first driver motor and second driver motor block in the Simulink program are applied to provide the input voltage to the actuator as written in Equation (2). After determining the position of the EMG sensor and the movement pattern to be measured, the next step is to set up a program on Simulink so that the EMG sensor can be integrated with the PI control program created on the wireless remote block before. In this step, changing the input to the EMG sensor is only necessary. The Simulink block “PID_EMG.slx” used for the EMG sensor is shown in Fig. 6(d). The block diagrams shown in Fig. 6(a), Fig. 6(c), and Fig. 6(d) are embedded to the Arduino Nano using Simulink Support Package for Arduino Hardware. A digital low-pass filter is applied to reduce the noise generated by the EMG signal output in the “EMG input” block (Fig. 6(d)). The filter is expressed in Equation (3) and applied in the Simulink block diagram as shown in Fig. 6(e). The complete guide for embedded control on the Arduino microcontroller using the Simulink Support Package for Arduino Hardware can be seen in a detailed manner on the MathWorks website [34].

(3) $$G\left(z\right)=\frac{0.06449}{z-0.9355}$$

## 6 Operation instructions

The operation instructions to embed the PI control block diagram are presented in Section 5.3. This section consists of the operation of the soft glove consists of two parts, how to wear the soft glove and how to operate the soft glove.

### 6.1 Wearing the soft glove instructions

**Fig. 7:**
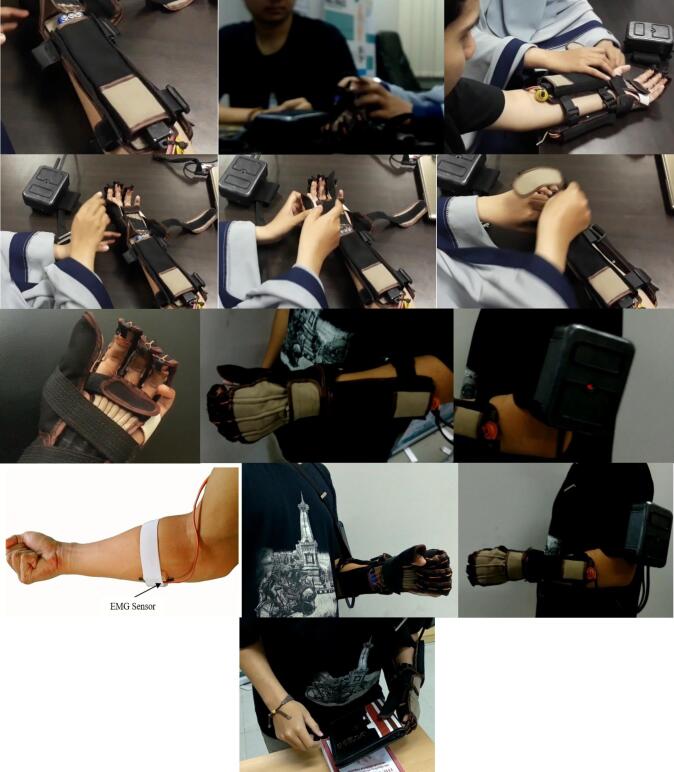
Attachment of portable soft exoskeleton glove to the user's hand.

The developed fabric-based soft exoskeleton glove is intended to be easy to attach and detach to the user's hand, as well as easy for the user to operate. The first step in wearing a soft glove is to wear the actuator base (Fig. 2(d)) on the user's upper which is between the wrist and elbow. The finger on the soft glove is worn to the user's fingers (index, middle, and ring). After the finger and actuator base are attached to the user, the binder on the actuator base is tightened. The user's thumb is adjusted so that the user can make a gripping motion when the soft glove gives full flexion assistance. The binder on the thumb is tightened so that it forms a certain fixed angle. After the soft glove has been placed on the user's hand, the control box is attached to the upper arm between the shoulder and elbow. The control box fastener can be tightened after the user is comfortable with the control box attached. For users who cannot move their elbows, the soft glove is equipped with straps so that the user's elbows can be adjusted to a certain angle so that the user feels comfortable holding and lifting objects. If the user wants to use the EMG as the input device, the soft glove is worn on the left hand while the EMG is attached to the right hand. The EMG is attached to the flexor carpi radialis muscle of the user's right hand. The user can provide commands by grasping the hand and then performing wrist flexion and extension. After the soft glove is attached to the user, the soft glove is ready to be operated. The photos of wearing the soft glove are presented in Fig. 7. The detailed instructions for wearing the soft glove can be seen in the video attachment “Movie Soft Exoskeleton Glove.mp4″.

### 6.2 Soft glove operation instructions

**Fig. 8:**
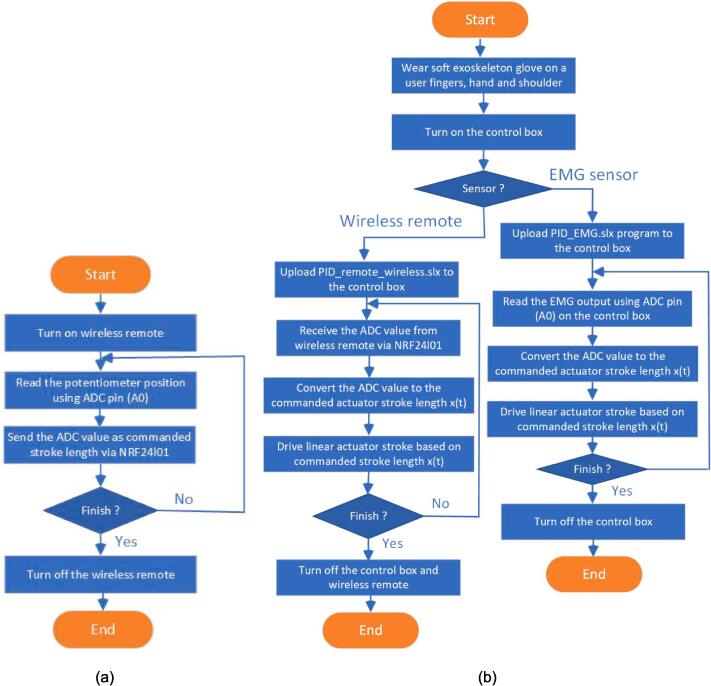
Operational flowchart of the soft glove (a) Wireless remote (b) Exoskeleton glove.

After the user has used the soft glove and control box, the operation of this soft glove is quite easy. On the sensor that will be used, the user can choose two sensors: the wireless remote (potentiometer) or the EMG sensor. The user can use the wireless remote if the user can no longer perform muscle contraction movements in the flexor carpi radialis muscle. Users can move the soft glove by rotating the wheel on the wireless remote with their left hand or with their big toe. If the user can still move the flexor carpi radialis muscle, EMG sensor is utilized. The EMG sensor is attached to the flexor carpi radialis. The flowchart of the soft glove can be summarized in Fig. 8.

## 7 Validation and characterization

**Fig. 9:**
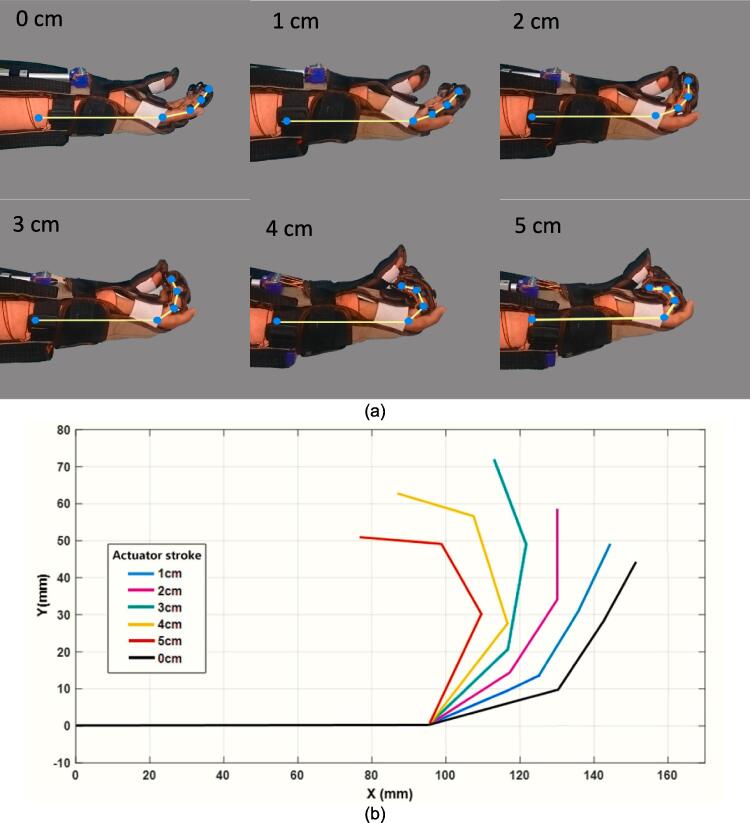
(a) Joint angle during finger flexion (b) Plot of joint angles of the finger.

In the soft glove test, the glove was attached to a normal healthy hand study participant. Soft glove movement testing is carried out to observe the curvature of the fingers that occurs in the soft glove from full extension to full flexion. The test is carried out by pulling the tendon 1 cm from the 0 cm position to the 5 cm position. Then the image of the change in the position of the ring finger is taken which is assumed to represent the position of the index finger and middle finger. The result of the user's fingers moving from flexion to extension is presented in Fig. 9. Based on the test, the developed soft glove can provide mechanical assistance to the fingers from rest poses to full flexion. Therefore, a user can grasp and hold various objects.

**Fig. 10:**
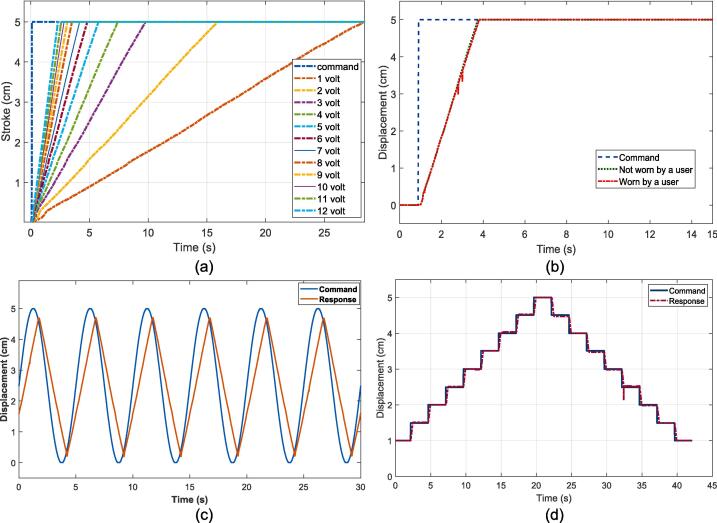
Closed loop responses of soft glove (a) Various given voltages to the linear actuator (b) Step input (c) Sinusoidal input (d) Multi-step input.

In this test, the actuator will be given commands to move the soft glove and test the performance of the PI control. In the first test, the PI control is given with an operating voltage ranging from 1 V to 12 V. Based on the voltage operation and time response graphs shown in Fig. 10 (a), it can be seen that the higher the operating voltage given to the linear actuator, the faster the resulting response time. Therefore, a working voltage of 12 Volts was chosen for the operating voltage in the soft glove. Furthermore, the step input command is given to determine the performance of the PI control. Results in Fig. 10 (b) shows the results of the plot of the command signal represented by the blue dash line, the response signal when the soft glove is not worn is represented by the green line, and the response signal when the soft glove is worn by the user is represented by the red dot line. Step input is applied to measure the time constant (τ) of the embedded PI control. This process is repeated three times. The repeated test produces the same result for the stroke displacement response from the step input, as depicted in Fig. 10(b). The time constant is measured based on Fig. 10(b). It is obtained from 63 % of the displacement final value (5 cm) at t = one time constant 1 τ. Based on Fig. 10(b), the obtained time constant is 1.76 s. The time constant is not fast but suitable enough to provide mechanical assistance to users from full flexion to full extension. Sinusoidal input command Fig. 10. (c) and multi-step Fig. 10(d) are given to test the performance of the PI on the trajectory following test. The results show that the actuator in the soft glove can follow sinusoidal and multi-step commands.

**Fig. 11:**
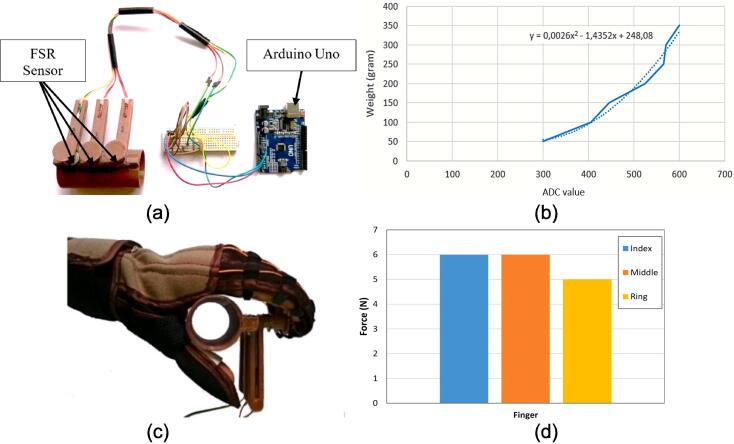
Measurement of generated force on each finger (a) Developed force instrument (b) FSR calibration result (c) Assisted force measurement (d) Measured force on each finger.

**Fig. 12:**
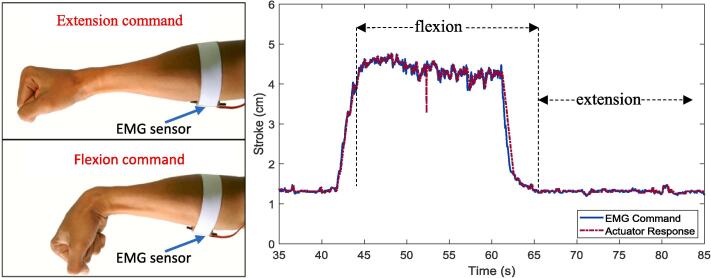
Response to the EMG signal input.

To test the force generated by the actuator when the soft glove provides mechanical support, three force sensitive resistors (FSRs) placed in the custom 3D printed structure were used to measure the generated force on the user's fingers. The photo of the developed instrument is shown in Fig. 11(a). The outputs for first, second, and third FSRs were connected to the Arduino Uno ADC pins i.e. A0, A1, and A2. Each FSR was connected to a 2.2 K Ω resistor. The FSR was calibrated with a digital scale to obtain the values in the gram unit. The calibrated result of the FSR is revealed in Fig. 11(b). The gram unit was scaled into Newton unit. The FSR sensor is placed on the surface of the 3D printer structure, and then the user's fingers make a gripping movement with a soft glove which is driven by a linear actuator as shown in Fig. 12(c). The actuator was commanded to pull the soft glove from 1 cm to 5 cm. The results of measuring the force generated by the soft glove can be presented in Fig. 11(d). The result is that the maximum force obtained is 6 N. This generated force has been reduced by tendon transmission and friction from the motor to the soft glove in the tendon hose.

In this test, the EMG sensor was attached to the flexor carpi radialis muscle of the user's hand. The position of the EMG sensor is shown in Fig. 12. The movements measured to obtain the EMG values are flexion and extension movements. The value read by the EMG sensor on the two types of movement will be used as an input value like the value given by the potentiometer on the wireless remote. After that, testing the performance of using soft gloves was also carried out using input from the EMG sensor. Because the EMG value obtained has quite a lot of noise. A low-pass filter was applied to reduce the EMG signal noise as shown in Equation (3) and Fig. 6(e). A low-pass filter (LPF) was applied to reduce the EMG signal noise. The results of performance testing between the input/command from the EMG sensor and the output/response of the linear actuator are presented in Fig. 12. The LPF was implemented to reduce the low-frequency noise or unwanted oscillation from the measured EMG signal during flexion/extension motion. Therefore, the cut-off frequency was selected with a low value at 3.33 rad/s or 0.53 Hz. The LPF was programmed using the “Discrete Transfer Function” block in Simulink under the “EMG input” block as depicted in Fig. 6(d). This LPF block was embedded in the Arduino using the Simulink Support Package for Arduino Hardware. The results of performance testing between the input/command from the EMG sensor and the output/response of the linear actuator are presented in Fig. 12.

Industry-grade medical sensor of electromyography (EMG) is not implemented as the input sensor. An affordable EMG sensor is selected as the EMG input to drive the soft exoskeleton. It does not output the raw value of EMG signals (oscillating signal and random). Instead, an amplified, rectified, and smooth signal is generated from this sensor as shown in Fig. 12 (right side). This output signal can be easily read using an ADC pin on the Arduino microcontroller. It is effective enough for measuring and monitoring muscle activation (contraction/relaxation or flexion/extension) and it is suitable for the input of prosthetic devices.

The output of the EMG signal from the “EMG analog input” block is an integer datatype, and it is converted into double. A digital low-pass filter as expressed in Equation (3) is applied to reduce the noise on the EMG output. Based on the measurements, the lowest value (in ADC) of the EMG signal is obtained during extension motion at the value of 300. While the highest value of the signal is received during flexion motion at the value of 500. Therefore a “Saturation“ block is implemented to ensure that the obtained signal has a value between 300 and 500. This output signal is then processed using a linear function as shown in Fig. 6(e). The output from this function ranges from 0 to 1023. This output value is mapped based on the linear actuator stroke length from 0 cm to 5 cm. A user can command the exoskeleton glove to move the stroke for 0 cm and 5 cm by performing extension and flexion commands respectively as shown in Fig. 12. The results show that the soft glove can follow commands from a given EMG signal. This test shows that in addition to the wireless remote (potentiometer), a user can utilize the EMG sensor to drive the soft glove and provide assistance with finger flexion and extension movements to grasp and hold various objects.

**Fig. 13:**
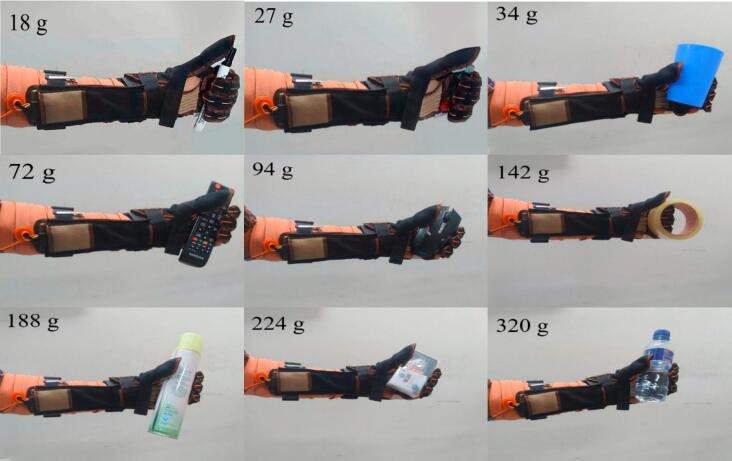
Various grasping tasks in daily activity.

To find out how far the soft glove can adapt when grasping and holding various shapes of given objects, a test was carried out to grasp and hold various objects. In this test, the wireless remote is used by the user to give commands to the soft glove. The test is carried out by giving the object to the soft glove that is being worn by a user, then the soft glove is driven from a neutral/rest state until it reaches a fully grasped state/full flexion. The hand-held objects used have cylindrical, beam, hollow cylinder, and irregular shapes. The result comparison of the test results is presented in Fig. 13 which shows movement testing by carrying out activities in the form of grasping and holding movements of various forms of objects. Based on the test results, the soft glove can be used to hold various shapes of objects, both small and large objects. Soft gloves are able to lift objects without falling with a weight of around 20 g to 320 g. These results indicate that the developed soft glove can provide mechanical assistance to grip various objects without falling. The resulting soft glove can be manufactured easily and quickly with materials found in e-commerce stores with relatively low production costs. In addition, soft gloves are also portable and lightweight, so that users can wear soft gloves comfortably and without fatigue for a long period of use. Othe various grasping tests can be seen in the video attachment “Movie Soft Exoskeleton Glove.mp4″.

## CRediT authorship contribution statement

**Rifky Ismail:** Writing – review & editing, Methodology, Conceptualization. **Mochammad Ariyanto:** Writing – original draft, Validation, Data curation. **Joga D. Setiawan:** Visualization, Investigation. **Taufik Hidayat:** Software, Investigation. **Paryanto:** . **Limbang K. Nuswantara:** Visualization, Supervision.

## Declaration of competing interest

The authors declare that they have no known competing financial interests or personal relationships that could have appeared to influence the work reported in this paper.
