# Supplementary material for: Design and testing of fabric-based portable soft exoskeleton glove for hand grasping assistance in daily activity
Source: HardwareX. 2024 May 7;18:e00537. doi: 10.1016/j.ohx.2024.e00537 (PMC11111837; doi:10.1016/j.ohx.2024.e00537)

Fabric-Based Portable Soft Exoskeleton Glove

A DESIGN

Glove

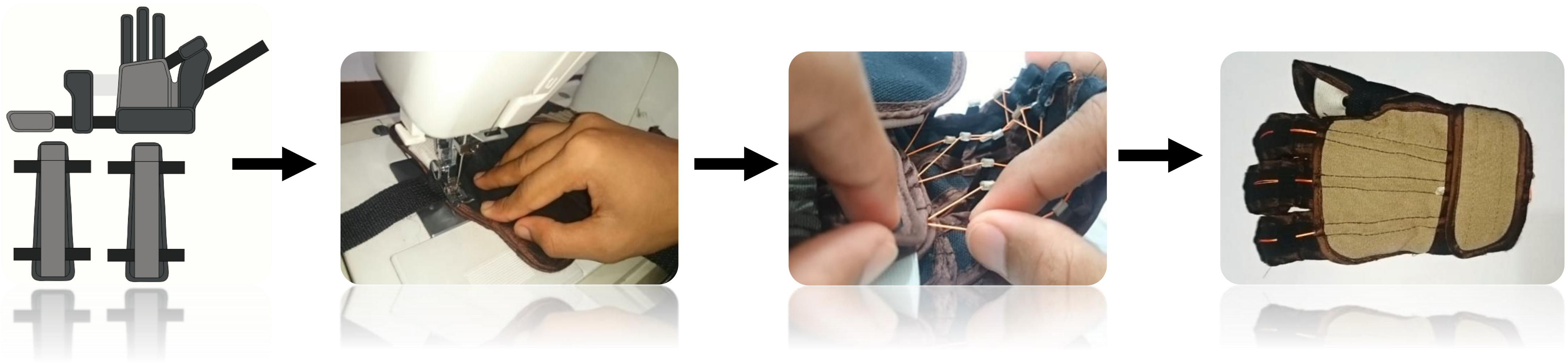

Controller box and Remote control

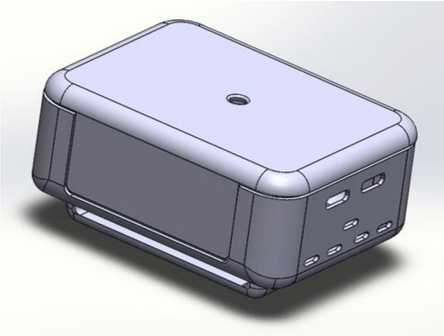

CAD Design
Controller box

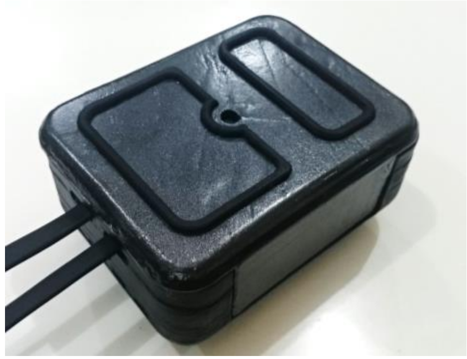

Finished product:
Controller box

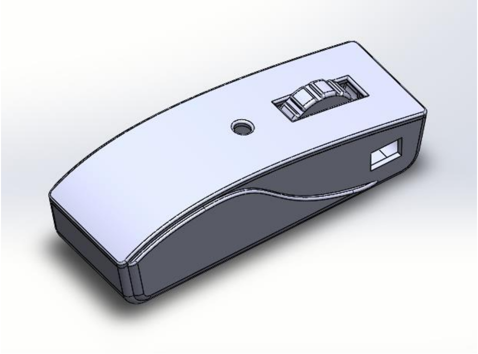

CAD Design
Remote control

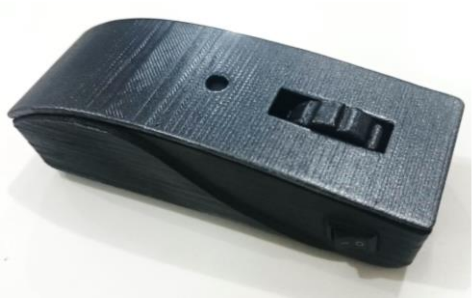

Finished product:
Remote control

Exosketeleton Glove

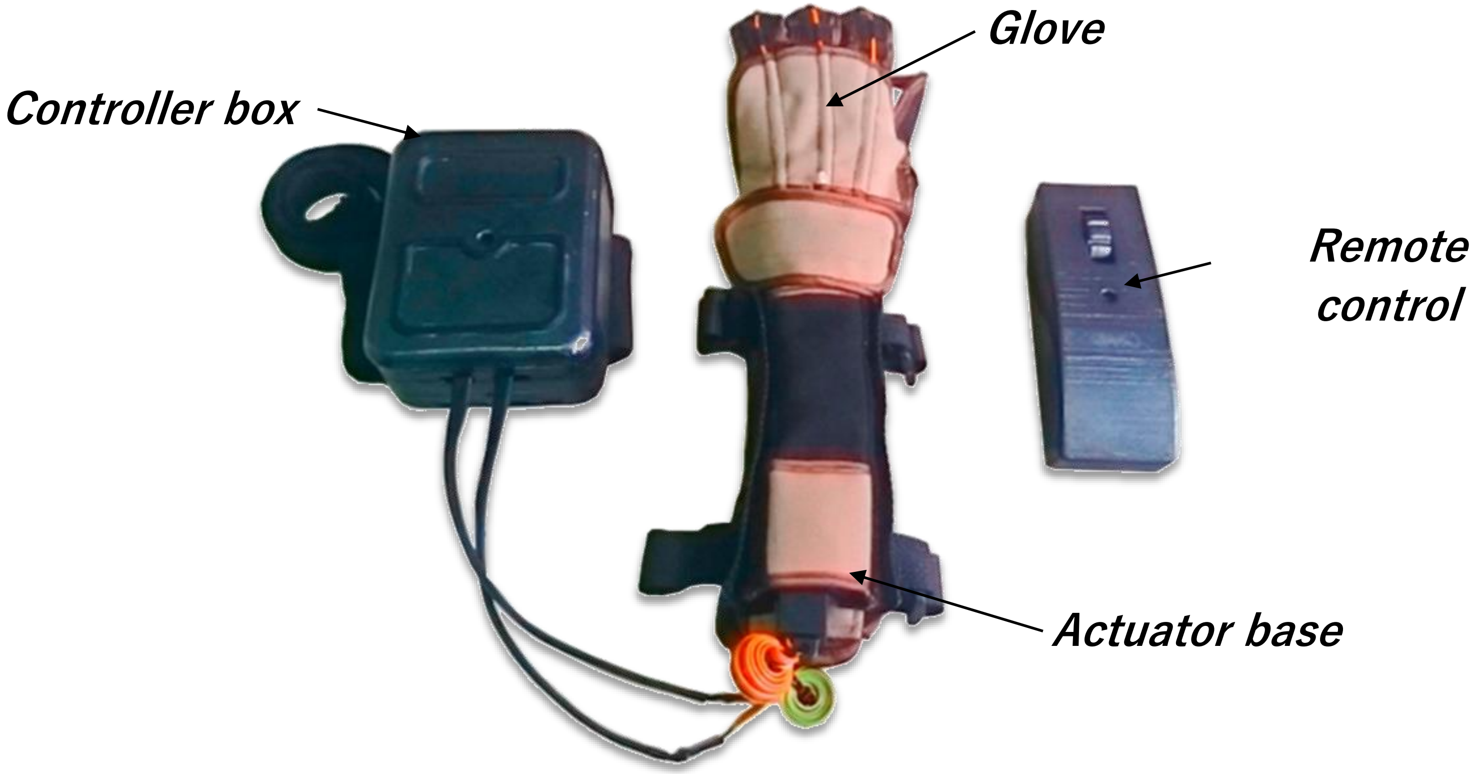

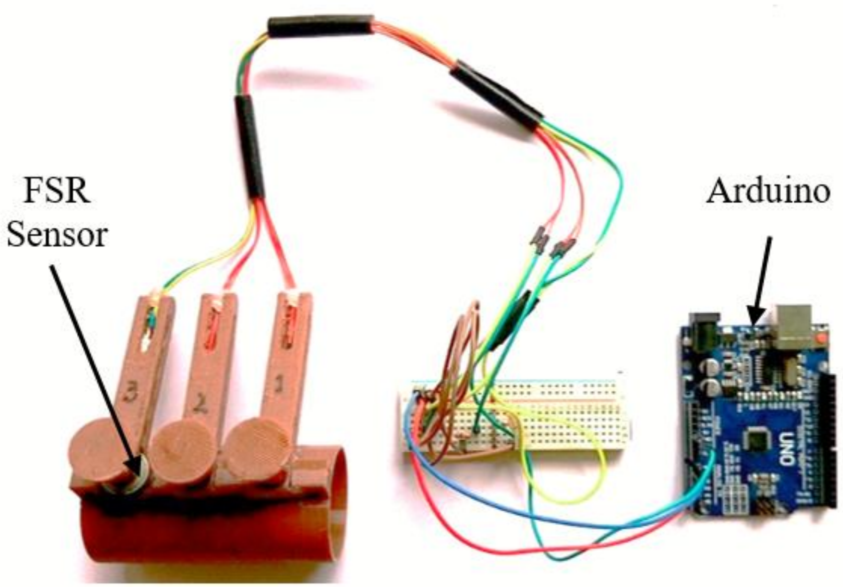

Supplement: Supplementary Data 1 [file mmc1.pdf]
